# Fat–Fit Patterns, Drug Consumption, and Polypharmacy in Older Adults: The EXERNET Multi-Center Study

**DOI:** 10.3390/nu13082872

**Published:** 2021-08-21

**Authors:** David Navarrete-Villanueva, Eva Gesteiro, Alba Gómez-Cabello, Asier Mañas, Rufino Pedro Olivares, José-Gerardo Villa-Vicente, Narcís Gusi, Marcela González-Gross, Ignacio Ara, Germán Vicente-Rodríguez, José Antonio Casajús

**Affiliations:** 1 GENUD (Growth, Exercise, NUtrition and Development) Research Group, University of Zaragoza, 50009 Zaragoza, Spain; dnavarrete@unizar.es (D.N.-V.); agomez@unizar.es (A.G.-C.); joseant@unizar.es (J.A.C.); 2 Department of Physiatry and Nursing, Faculty of Health Sciences (FCS), University of Zaragoza, 50009 Zaragoza, Spain; 3 Red Española de Investigación en Ejercicio Físico y Salud, EXERNET, University of Zaragoza, 50009 Zaragoza, Spain; asier.manas@uclm.es (A.M.); ngusi@unex.es (N.G.); marcela.gonzalez.gross@upm.es (M.G.-G.); ignacio.ara@uclm.es (I.A.); 4 Instituto Agroalimentario de Aragón-IA2-(CITA-Universidad de Zaragoza), 50009 Zaragoza, Spain; eva.gesteiro@upm.es; 5 ImFine Research Group, Department of Health and Human Performance, Universidad Politécnica de Madrid, 28040 Madrid, Spain; 6 Centro Universitario de la Defensa, University of Zaragoza, 50090 Zaragoza, Spain; 7 Centro de Investigación Biomédica en Red de Fisiopatología de la Obesidad y Nutrición (CIBEROBN), 28029 Madrid, Spain; 8 GENUD Toledo Research Group, Universidad de Castilla-La Mancha, 45071 Toledo, Spain; 9 CIBER of Frailty and Healthy Aging (CIBERFES), 28040 Madrid, Spain; 10 Physical Activity and Quality of Life Research Group (AFYCAV), Faculty of Sport Sciences, University of Extremadura, 10003 Cáceres, Spain; olivares.pedro@gmail.com; 11 Faculty of Education, Psychology and Sport Sciences, University of Huelva, Avenida de las Fuerzas Armadas s/n, 21007 Huelva, Spain; 12 Grupo de Investigación VALFIS, Instituto de Biomedicina (IBIOMED), Facultad de Ciencias de la Actividad Física y del Deporte, Universidad de León, 24071 León, Spain; jg.villa@unileon.es; 13 Department of Physiatry and Nursing, Faculty of Health and Sport Sciences (FCSD), University of Zaragoza, 22002 Huesca, Spain; 14 Department of Physiatry and Nursing, Faculty of Medicine, University of Zaragoza, 50009 Zaragoza, Spain

**Keywords:** physical fitness, fatness, older populations, polypharmacy, drug consumption

## Abstract

Background: Physical fitness levels and the amount of accumulated adipose tissue (fatness) relate to current and future individuals’ heath status. Nevertheless, the interrelationships of their combined patterns with polypharmacy and the types of medications consumed have not been sufficiently investigated. Methods: This cross-sectional study was carried out in six Spanish regions between 2008 and 2009 with a sample of older community-dwelling adults (≥65 years old) without dementia or cancer. Fitness was measured with one-leg balance and senior fitness tests, as well as by measuring weight and fat mass with a bioelectrical impedance analyzer. Polypharmacy was defined as the use of five or more medications. An analysis of variance was performed for comparisons between the physical fitness and fatness patterns and the medication consumed. Results: A total of 1709 elders were included in the study (72.1 ± 5.2 years). The two unfit patterns were those with the highest drug consumption. The High-Fat–Unfit pattern was the one that had the most significant consumption and had the highest percentage of polymedicated subjects. The Low-Fat–Fit pattern had a significantly lower percentage of people that did not consume any medications. The highest percentages of drug consumption in 7 of the 10 groups that were included were concentrated in the two unfit patterns. Conclusions: This study highlights the importance of fitness in older adults, as it is at least as important as the avoidance of accumulation of excess fat with respect to the consumption of a smaller number of medicines.

## 1. Introduction

The physical fitness level and the amount of accumulated adipose tissue (fatness) are both sensitive and specific biomarkers of physiological decline in older adults [1]. Low fitness and high or very low fatness conditions are independently associated with adverse health outcomes in older adults, such as functional impairment, hospitalization, falls, and morbidity [2,3,4].

On the other hand, aging is also associated with an increased risk of experiencing more than one chronic condition at the same time [5,6]. The impacts of multimorbidity on an older person’s capacities, healthcare utilization, and cost of care are often significantly greater than what might be expected from the summed effects of each condition [7]. In relation to this, older people usually take more medications than younger people, and they often take several medications at the same time, which is known as ‘polypharmacy’ [5].

Drug consumption not only implies protective effects, but also negative ones that are derived from drug–drug, drug–food, and drug–disease interactions, side effects, and/or inappropriate dosages of medications [8]. In this sense, polypharmacy—defined as co-prescribing more than five medications [9]—negatively affects patient care by potentially increasing both healthcare costs and the risk of health-related issues, including falls, hospital readmission, longer hospital admissions, adverse drug reactions, and mortality [9,10,11].

Polypharmacy is, in fact, a common health concern among older adults. It ranges from around 4% to about 96.5% depending on the age group, definition, healthcare setting, and region [12]. A cross-sectional analysis from database of “wave 6” of the Survey of Health, Ageing, and Retirement in Europe, which included data from 34,232 older people, revealed that the prevalence of polypharmacy ranged from 26.3% to 39.9% across 17 European countries [13].

In this specific area of pharmacology, there is clear evidence of bidirectional associations of low physical fitness and high fatness with polypharmacy and medication consumption [14,15]. Although it is widely known that obesity and overweight (present in 84% of the older population), as well as low physical fitness, have a profound impact on health [3,15,16], not many studies have investigated their interaction with negative health outcomes.

In this regard, the popularly conceptualized “fat-but-fit” theory, which is explained by the idea of it being “better to be fat and fit than low-weight and unfit”, is being increasingly studied [1]. There is emerging evidence suggesting that improved physical fitness might counteract the negative consequences of high adiposity with respect to mortality risk [17,18]. However, little is known about the interactions of fitness, fatness, and drug consumption.

To improve the understanding of the association of the cooccurrence of low fitness and fatness in older adults with drug consumption, it is necessary to identify Fat–Fit patterns and to explore their relationships with polypharmacy and the types of medication consumed. To the best of our knowledge, there are no studies that have addressed this relationship among older adults by using cluster analysis and including both physical fitness tests and adiposity measures.

Therefore, the first aim of the present study was to determine the association between Fat–Fit patterns in the elderly and polypharmacy, the number of medications, and the lack of drug consumption. The second aim was to explore the relationship between Fat–Fit patterns and the types of medication consumed.

## 2. Materials and Methods

### 2.1. Study Design and Participants

This cross-sectional study was part of the EXERNET Multi-Center Study; it was carried out in six Spanish regions between 2008 and 2009. The overall project was aimed at developing reference values for physical fitness and body composition in a representative sample of older Spanish community-dwelling adults and determining their associations with several health outcomes. The complete methodology of this project was published previously [16].

A representative sample of community-dwelling older adults was used; participants were eligible for enrollment if they fulfilled the following criteria: (1) They were aged over 65 years; (2) they were non-institutionalized people; and (3) they were not suffering from dementia or cancer.

Of an initial total sample of 3093 participants, 2299 performed all of the physical fitness assessments. Of these, 1709 answered the medication questionnaire and were selected for the present study.

### 2.2. Ethics

The study protocol received ethical approval from the Clinical Research Ethics Committee of Aragón (Spain) (18/2008), and it adhered to the Helsinki Declaration of 1964 (revision of Edinburgh (2000) and further amendments). All participants provided written informed consent before they were enrolled in the study.

### 2.3. Measurements

#### 2.3.1. Physical Fitness and Body Composition

Anthropometric and physical fitness measures were performed by trained researchers according to standardized protocols after a workshop training session in order to standardize and harmonize the assessments [19].

In order to assess body composition, height was measured by using a portable stadiometer (Seca, Hamburg, Germany) with a maximum capacity of 2.10 m and a an error margin of 0.001 m. Participants stood barefoot with their scapula, buttocks, and heels resting against a wall; the neck was held in a natural, non-stretched position, the heels were touching each other with the tips of the toes spread to form a 45° angle, and the head was held straight with the inferior orbital border in the same horizontal plane as the external auditory tube (Frankfort’s plane) [20]. Body weight and body fat percentage (%BF) were measured with a portable bioelectrical impedance analyzer (Tanita BC 418-MA (Tanita Corp., Tokyo, Japan)) with a maximum capacity of 200 kg and an error margin of ±100 g without shoes, socks, or heavy clothes. Body mass index (BMI) was calculated as weight (in kilograms) divided by the square of the height (in meters). Before the examinations, the study subjects received the following recommendations: (1) no alcohol less than 12 h prior to measurement, (2) no vigorous exercise less than 12 h prior to measurement, (3) no excess food or drink on the day before measurement, (4) no food or drink less than 3 h prior to measurement, and (5) urination immediately before measurement.

The physical fitness measurements included the senior fitness test battery designed by Rickly and Jones [21] with the following physical fitness components: lower- and upper-body strength measured with the chair-stand test and arm-curl test, respectively, agility/dynamic balance measured with the eight-foot up-and-go test, and cardiorespiratory fitness (CRF) measured with the 6-min walk test. In addition, the static balance was assessed with the one-leg test [22]. All of the tests were performed twice, except for the chair-stand test and the 6-min walk test, which were performed once [23].

#### 2.3.2. Fat–Fit Patterns

**Figure 1:**
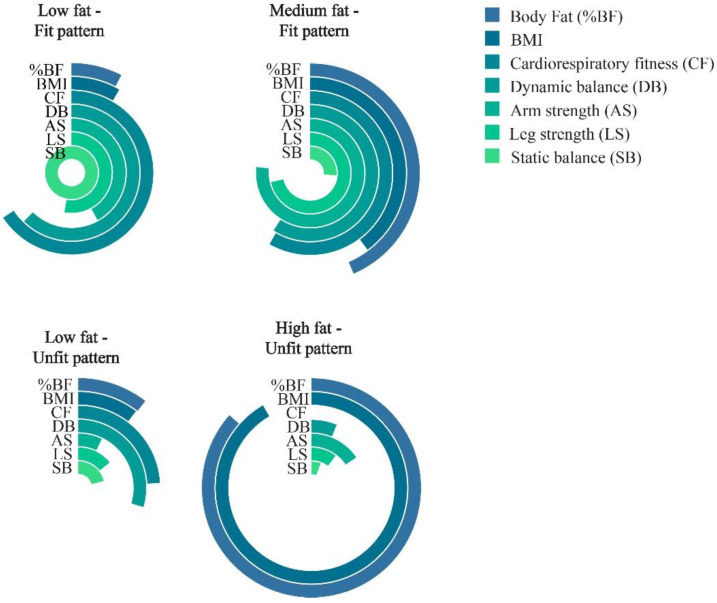
Physical fitness and body fat z-scores, adjusted by sex and age. Note: The z-scores were reconverted into units from 0 to 1 to create this figure.

The Fat–Fit patterns, which were developed with the cluster methodology and published elsewhere [24], were used to classify the study subjects into four groups according to their fatness and physical fitness levels, taking age and sex into account. The four Fat–Fit patterns created (Figure 1) were the following:

- Low-Fat–Fit pattern (LFat-Fit), which was characterized by high levels of fitness, especially for the balance test, and the lowest levels of BMI and %BF.
- Medium-Fat–Fit pattern (MFat-Fit), which included the subjects with the highest values for strength, high levels of dynamic balance and CRF, and the presence of medium values for both of the body composition variables studied.
- High Fat–Unfit pattern (HFat-Unfit), which included subjects with the lowest values for physical fitness and the highest values for BMI and %BF.
- Low-Fat–Unfit pattern (LFat-Unfit), which included subjects with low values for the physical fitness variables and low values for both the BMI and %BF in comparison with the other groups.

#### 2.3.3. Demographic Characteristics

Individual interviews were performed by trained researchers to register sociodemographic questionnaires. The physical activity (PA) levels and sedentary behaviors of participants were assessed with the validated Elderly EXERNET Physical Activity Questionnaire (EEPAQ) [25]. These questions identified two types of activities: walking and sitting time. Organized physical activity (OPA) was also registered with the following question: “Are you currently engaged in organized physical activity?” This question registered any OPAs, which were understood as collective, guided, and supervised activities provided by an instructor. To measure smoking habits, the question “Do you currently smoke?” (no/yes) was used to classify the subjects as smokers or non-smokers.

#### 2.3.4. Polypharmacy and Medicine Consumption

The names of medications, frequency of consumption, and ingested doses were collected in telephone calls by trained researchers after the fieldwork. For the purpose of drug analysis, we included the 10 most consumed drug groups in Spain in the statistical analysis (Table 1); these were classified by the Anatomical Therapeutic Chemical (ATC) classification system, which discriminates among medicines according to composition, mechanism of action, and main applicability [26,27]. Polypharmacy was defined by the concomitant consumption of ≥5 medications within the 30 days prior to the interview [9].

### 2.4. Statistical Analyses

Descriptive data were reported as means (SD) for continuous variables and frequencies (percentages) for categorical data. The four Fat–Fit patterns included in this study were previously created with a cluster analysis; a detailed description of the process was published elsewhere [24]. Clustering techniques are a form of unsupervised learning that gathers elements in homogeneous groups based on the similarities between them. A z-test for proportions was performed in order to verify the statistical significance of the types of drugs consumed for each Fat–Fit pattern. An analysis of variance (ANOVA) with Bonferroni correction was performed for comparisons between multiple patterns and the numbers of medications consumed. Statistical significance was set at *p*-value < 0.05. The IBM SPSS (SPSS Inc., Chicago, IL, USA) software (version 26) was used for the statistical analysis.

## 3. Results

### 3.1. Participants’ Characteristics

The descriptive characteristics according to the Fat–Fit patterns are presented in Table 2 and in the Electronic Supplementary Material. A total of 1709 older adults were included in the study, with a mean baseline age of 72.1 ± 5.2 years.

### 3.2. Relationships between Fat–Fit Patterns, Number of Medications Consumed, and Polypharmacy

**Figure 2:**
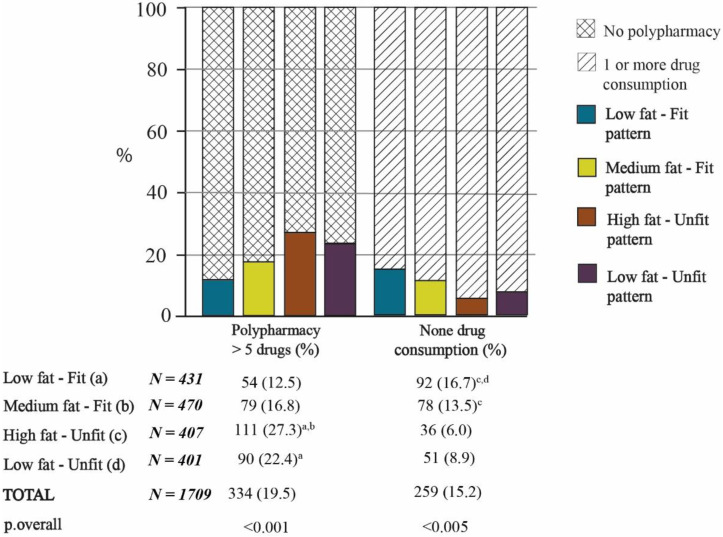
Prevalence of polypharmacy and of lack of drug consumption according to the Fat–Fit patterns. Note: The statistical significance of the proportions between groups can be observed in the columns. The letters in superscript indicate the bilateral comparisons that are significantly different (*p* < 0.05).

Polypharmacy concerned 19.5% of the study population (*n* = 334). Figure 2 illustrates the proportions of polypharmacy in each Fat–Fit pattern. The rate of polypharmacy was significantly higher in the HFat-Unfit pattern than in the fit patterns (*p* < 0.05, Figure 2). In addition, the proportion of polypharmacy was significantly greater for the LFat-Unfit pattern for the LFat-Fit pattern (*p* < 0.05, Figure 2). On the other hand, 15.2% of the sample did not consume any medications. The LFat-Fit pattern had a significantly lower percentage of people that did not consume any medications compared to the two unfit patterns (*p* < 0.05, Figure 2).

Regarding the total number of medications, the average was 2.75 ± 2.13. As shown in Table 2, older adults belonging to the HFat-Unfit pattern took significantly more medicines than those that belonged to the MFat-Fit and the LFat-Fit patterns (both *p* < 0.001), as well as the LFat-Unfit pattern (*p* = 0.036). Similarly, people belonging to the LFat-Unfit pattern took significantly more medicines than those belonging to the LFat-Fit pattern (*p* < 0.001).

### 3.3. Relationships between Fat–Fit Patterns and Drug Groups

#### 3.3.1. Fat–Fit Patterns and Drugs Related to the Alimentary Tract and Metabolism

**Figure 3:**
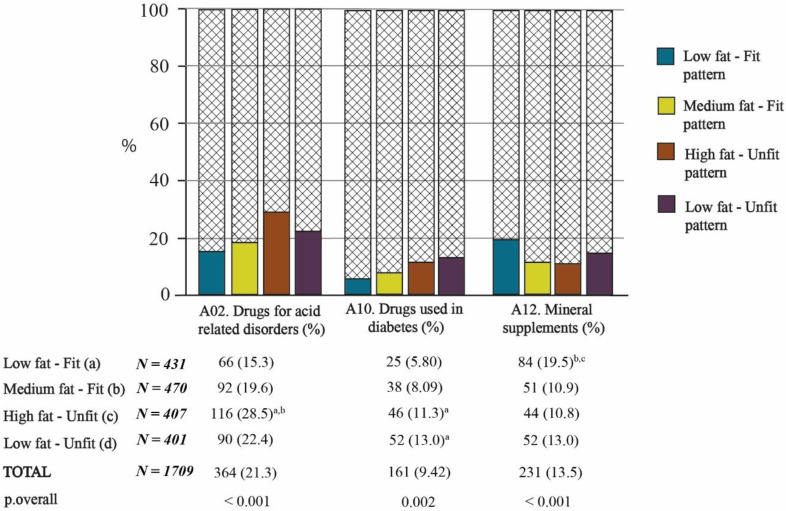
Consumption of drugs related to the alimentary tract and metabolism according to the Fat–Fit patterns. The statistical significance of the proportions between groups can be observed in the columns. The letters in superscript indicate the bilateral comparisons that are significantly different (*p* < 0.05).

According to the ATC classification system, within the group of drugs classified being related to the “alimentary tract and metabolism” (Group A), there were three subgroups with a high prevalence of use in the elderly population studied: drugs for acid-related disorders (A02), drugs used in diabetes (A10), and mineral supplements (A12), which were taken by 21.3%, 9.42%, and 13.5% of the sample, respectively. Their distributions across the Fat–Fit patterns were also different (*p* < 0.05, Figure 3).

Considering the Fat–Fit patterns, the use of drugs for acid-related disorders was significantly higher in the HFat-Unfit pattern compared with both of the fit patterns. Similarly, there was a significantly higher proportion of consumption of drugs from group A10 for both unfit patterns in comparison with the LFat-Fit pattern. Regarding the mineral supplement group, there was a significantly higher proportion of use in the LFat-Fit pattern than in the MFat-Fit and HFat-Unfit pattern (all *p* < 0.05, Figure 3).

#### 3.3.2. Fat–Fit Patterns and Drugs Related to the Cardiovascular System and Blood

**Figure 4:**
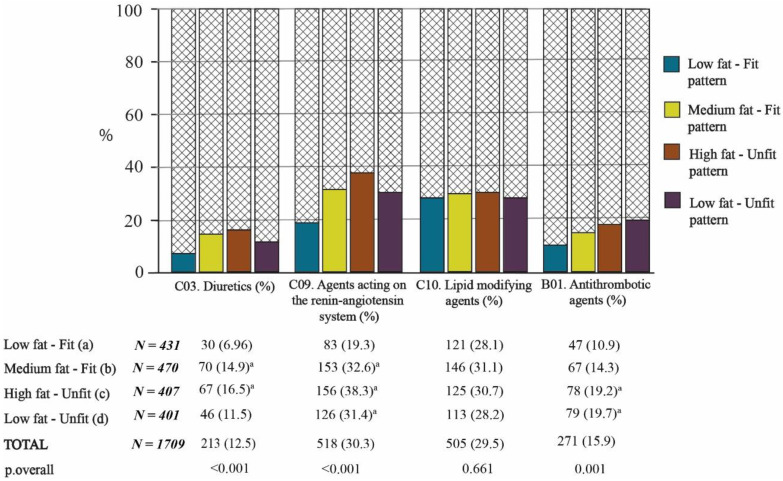
Consumption of drugs related to the cardiovascular system and blood according to the Fat–Fit patterns. The statistical significance of the proportions between groups can be observed in the columns. The letters in superscript indicate the bilateral comparisons that are significantly different (*p* < 0.05).

Figure 4 shows that there was a significantly higher proportion of use of diuretics (C03) and agents acting on the renin–angiotensin system (C09) in the fat pattern (MFat-Fit and HFat-Unfit) in comparison with the LFat-Fit pattern (*p* < 0.05). Additionally, the subjects belonging to the Low-Fat–Unfit pattern consumed more drugs in the C9 group than the subjects belonging to the Low-Fat–Unfit one (*p* < 0.05, Figure 4). No significant differences between groups were observed for the consumption of blood-lipid-modifying agents (group C10).

Regarding the B1 group of drugs (antithrombotic agents), on average, the unfit groups had the highest intake, and this intake differed significantly from that of the LFat-Fit pattern (*p* < 0.05, Figure 4).

#### 3.3.3. Fat–Fit Patterns and Drugs Related to the Musculoskeletal and Nervous Systems

**Figure 5:**
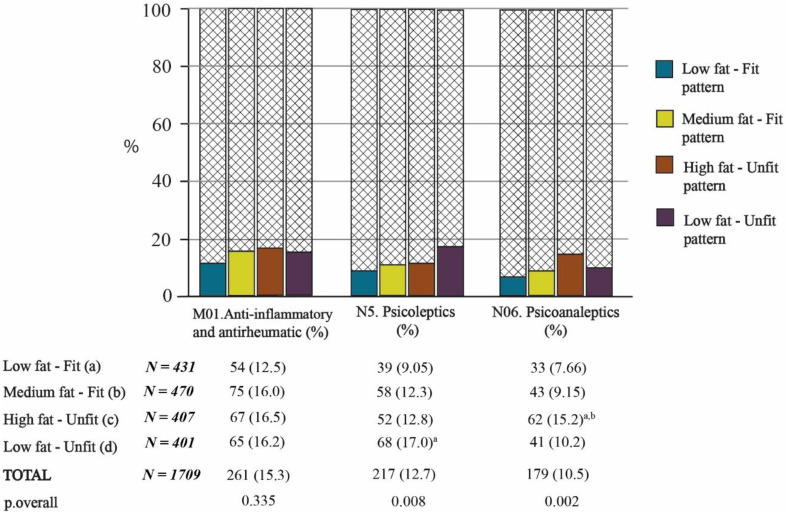
Consumption of drugs related to the musculoskeletal and nervous systems according to the Fat–Fit patterns. The letters in superscript indicate the bilateral comparisons that are significantly different (*p* < 0.05).

Regarding drugs related to the nervous system, there were differences between groups in the consumption of psycholeptics (N05) and psychoanaleptics (N06) (both *p* < 0.05, Figure 5). The highest proportion of consumption of psycholeptics was observed in the LFat-Unfit pattern, which differed significantly from the LFat-Fit pattern (*p* < 0.05, Figure 5). Nevertheless, the proportion of people who consumed psychoanaleptic drugs was higher in the HFat-Unfit pattern, which differed significantly from the two fit patterns (both *p* < 0.05, Figure 5). As with group C10, there were no significant differences in the consumption of anti-inflammatory and antirheumatic products (M01) between patterns (Figure 5).

## 4. Discussion

The present study was designed to analyze the relationship of fatness and physical fitness with the consumption of medication in older adults. To the best of our knowledge, this is the first study to consider physical fitness and fatness as unified parameters for the study of consumption of medications in older people.

The initial objective of this study was to identify the associations of the Fat–Fit patterns with the number of medications consumed, polypharmacy, and the lack of drug consumption. In this regard, the two unfit patterns (HFat-Unfit and LFat-Unfit), which were characterized in a previous study as those with the highest risk of mortality (HR:1.68 and HR:2.01, respectively) [24], were the two patterns with the greatest drug consumption. HFat-Unfit was found to significant the most (with significance) and had the highest percentage of polymedicated subjects (≥5 medicines) in comparison with the fit patterns.

These findings further support the results of previous studies that observed a bidirectional association between polypharmacy and physical fitness in older adults, especially in some components of physical performance, such as walking speed [28,29], strength [29,30], or static balance [30]. However, Volaklis et al. [31] found no differences in muscular strength between persons with and without polypharmacy, unlike Rawle et al. [30] and Sganga et al. [29]. It is likely that these differences could be explained by the high prevalence (25.3%) of joint diseases in the sample used by Volakilis et al. (patients with arthritis or rheumatism can exhibit less handgrip strength). Likewise, Verde et al. [32] found that agility (dynamic balance) was associated with risk of several potential drug interactions in polymedicated octogenarians, which may result in drug toxicity, reduced pharmacological effects, and adverse drug reactions.

Another significant aspect was that the findings for the MFat-Fit pattern were in line with other authors’ descriptions of the “fit-but-fat” paradigm [24]. This pattern was characterized by the highest values for strength, high levels of dynamic balance and CRF, and medium values for both body composition measures, which, on average, would be classified as overweight. The older adults that belonged to this pattern consumed significantly less medication than subjects included in the other fat pattern (HFat-Unfit) or did not take any medications at all (Figure 2). However, the difference between the two fat patterns was not only in the physical fitness level, but also in the fatness level, as the HFat-Unfit pattern had a significantly higher %BF value than the MFat-Fit group, as it is, on average, characterized as an obese pattern. In the same way, Assari et al. [14] found an association between obesity and polypharmacy, but very few studies have focused on the relationship between the co-prescription of multiple medications and body composition measurements.

It is interesting to note that subjects in the LFat-Fit pattern consumed significantly less medication. This group was the one with the lowest proportion of polypharmacy and included the highest percentage of subjects who did not take any medications. The mixture of low fatness and high fitness continues to be the most strongly linked with having fewer health risks [33,34]; this fact contrasts with the reality of the aging process, in which most people gain weight [35] and become less fit [36]. It is therefore urgent and important to incorporate all strategies that enable active and healthy aging.

Moving on to consider the type of drug consumption, the two unfit patterns were found to have the highest percentages of drug consumption for 7 of the 10 groups of drugs included in the study. The prescription of antiacids (A02) is probably due to the association of obesity with gastroesophageal reflux [37], while the use of psychotropics (N05 and N06), antidiabetics (A10), and antithrombotics (B01) may be linked to the development of pathologies related to low PA. Specifically, it is encouraging to compare our results with those of Loggia et al. [38], who found an independent association between psychotropic use and physical fitness (measured with the Timed Up and Go test).

The consumption of diuretic drugs (C03) and antihypertensive drugs (C09) was also more frequent in the older MFat-Fit and HFat-Unfit adults, as well as in LFat-Unfit group—specifically with respect to group C9—in comparison with the LFat-Fit pattern (Figure 4). This finding broadly supports the work of other authors in this area who linked obesity with consumption of both types of cardiac drugs, which is basically because obesity and overweight are common risk factors for heart disease [39,40,41]. A possible explanation for this might be that the MFat-Fit pattern had significantly lower CRF results and a higher proportion of people who consumed drugs from the C03 and C09 groups.

One unanticipated finding was that there were no differences between patterns regarding lipid-modifying agents (group C10) and anti-inflammatory and antirheumatic medicines (group M01), as both groups of drugs showed very homogeneous consumption levels at this age. This result may be explained by the fact that these groups are two of the most commonly consumed medicines in Spain [42]. There is evidence supporting that the use of the lipid-modifying agents in older people produces significant reductions in major vascular events, irrespective of age [43], which could lead to the very lax requirements for prescription of these drugs. Regarding the group of anti-inflammatory drugs, which are mainly used to manage pain, the abusive use of these drugs—without differences between groups—may be associated with the ability to buy them in pharmacies without the need for a doctor’s prescription.

Finally, another unexpected finding was that of the significantly high consumption of mineral supplements (A12) by the subjects belonging to the LFat-Fit pattern. A possible explanation for this result may be that the LFat-Fit group was associated with healthier habits, which would explain the higher consumption of mineral supplements. This finding is consistent with that of Aparicio-Ugarriza et al. [44] who found that the high-physical-fitness group showed a better micronutrient intake profile than those of other physical fitness groups, and the low-physical-fitness group showed an inadequate intake of some micronutrients, such as potassium, vitamin D, and vitamin E.

The limitations of the current study include the loss of participants due to the incompleteness of the data. Also, the cross-sectional design of the study does not allow the establishment of a cause–effect relationship between the Fat-fit patterns and medication consumption variables, so it is possible that those with comorbidity (and higher prevalence of drug use) may have reduced their PA level due to their disease status. Another limitation was the non-registration of the pathologies in the EXERNET multicenter study subjects; therefore, it is not possible to analyze which group has more pathologies, nor the prevalence of each of them in each group. There are also several strengths to report, such as the strict and validated protocol of the fieldwork and the assessment of physical fitness through different and specific physical fitness tests.

## 5. Conclusions

The present study highlighted the importance of physical fitness in older adults; it is at least as important as the avoidance of accumulation of excess fat with respect to the consumption of a smaller number of medicines and the potential negative interactions that these medicines could have in older community-dwelling adults.

The promotion of a high physical fitness level with specific exercise programs in this population could provide a better health status and, thus, reduce the need for the co-prescription of several drugs, therefore preventing the side effects of polypharmacy and promoting healthier and more sustainable aging at the individual and societal level.

## Figures and Tables

**Table 1:** The 10 most consumed drug groups in older Spanish adults.

	First-Level Group	Second-Level Group
1	A. Alimentary tract and metabolism	A02. Drugs for acid-related disorders
2		A10. Drugs used in diabetes
3		A12. Mineral supplements
4	B. Blood and blood-forming organs	B01. Antithrombotic agents
5	C. Cardiovascular system	C03. Diuretics
6		C09. Agents acting on the renin–angiotensin system
7		C10. Blood-lipid-modifying agents
8	M. Musculoskeletal system	M01. Anti-inflammatory and antirheumatic products
9	N. Nervous system	N05. Psycholeptics
10		N06. Psychoanaleptics

**Table 2:** Summary of the descriptive characteristics according to the baseline Fat–Fit patterns.

	TOTAL	Low-Fat–Fit Pattern	Medium-Fat–Fit Pattern	High-Fat–Unfit Pattern	Low-Fat–Unfit Pattern	p.overall
	*N* = 1709	*N* = 431	*N* = 470	*N* = 407	*N* = 401	
Age (year) (mean (SD))	72.1 (5.20)	71.1 (4.87)	72.7 (5.39)	72.1 (5.04)	72.4 (5.35)	0.001
Sex (female)	1300 (76.1%)	317 (73.5%)	361 (76.8%)	310 (76.2%)	312 (77.8%)	0.5
OPA (yes)	1487 (88.2%)	385 (90.6%)	424 (92.0%)	352 (88.0%)	326 (81.7%)	0.001
Walking hours per day						0.004
<1	506 (30.5%)	97 (23.1%)	132 (29.1%)	147 (37.7%)	130 (32.8%)	
1–2	882 (53.2%)	240 (57.1%)	243 (53.6%)	191 (49.0%)	208 (52.5%)	
2–3	216 (13.0%)	68 (16.2%)	60 (13.2%)	43 (11.0%)	45 (11.4%)	
3–4	34 (2.05%)	10 (2.4%)	11 (2.4%)	6 (1.5%)	7 (1.8%)	
4–5	10 (0.6%)	2 (0.5%)	4 (0.9%)	2 (0.5%)	2 (0.5%)	
>5	11 (0.7%)	3 (0.7%)	3 (0.7%)	1 (0.3%)	4 (1.0%)	
Sitting hours per day						0.004
<1	40 (2.5%)	12 (2.9%)	7 (1.6%)	10 (2.6%)	11 (2.9%)	
1–2	154 (9.6%)	49 (12.0%)	40 (9.7%)	24 (6.3%)	41 (10.8%)	
2–3	449 (28.0%)	136 (33.3%)	108 (25.0%)	95 (24.9%)	110 (28.9%)	
3–4	417 (26.0%)	94 (23.0%)	110 (25.5%)	106 (27.8%)	107 (28.2%)	
4–5	272 (17.0%)	64 (15.7%)	89 (20.6%)	68 (17.8%)	51 (13.4%)	
>5	269 (16.8%)	53 (13.0%)	78 (18.1%)	78 (20.5%)	60 (15.8%)	
Smoking (%yes)	55 (3.3%)	18 (4.3%)	8 (1.8%)	12 (3.1%)	17 (4.3%)	0.07
Fat Mass (%)	37.1 (6.87)	33.6 (6.4)	37.9 (5.9)	42.5 (5.6)	34.6 (5.9)	<0.001
BMI (kg/m^2^)	29.2 (4.1)	26.5 (2.8)	29.2 (2.9)	33.9 (3.5)	26.9 (2.6)	<0.001
Number of Medicines (mean (SD))	2.75 (2.1)	2.23 (1.9)	2.61 (2.1)	3.27 (2.2) ^a,b,d^	2.96 (2.2) ^a^	<0.001

Note: Values bearing different superscript letters differ significantly (*p* < 0.05). OPA: Organized Physical Activity; N Med: Number of medications; BMI: Body Mass Index; SD: Standard deviation

## Data Availability

The data are not publicly available due to privacy.

## Supplementary Materials

The following are available online at https://www.mdpi.com/article/10.3390/nu13082872/s1, Figure S1: Distribution of age in the four Fat-Fit patterns, Figure S2: Distribution of sex in the four Fat-Fit patterns, Figure S3: Distribution of fat percentage in the four Fat-Fit patterns, Figure S4: Distribution of BMI in the four Fat-Fit patterns.
