# Supplementary material for: Fat–Fit Patterns, Drug Consumption, and Polypharmacy in Older Adults: The EXERNET Multi-Center Study
Source: Nutrients. 2021 Aug 21;13(8):2872. doi: 10.3390/nu13082872 (PMC8400623; doi:10.3390/nu13082872)

Electronic Supplementary Material 1. Distribution of age, sex, taking medication (polypharmacy), BMI and fat percentage in the Fat-Fit patterns

Figure S1. Distribution of age in the four Fat-Fit patterns

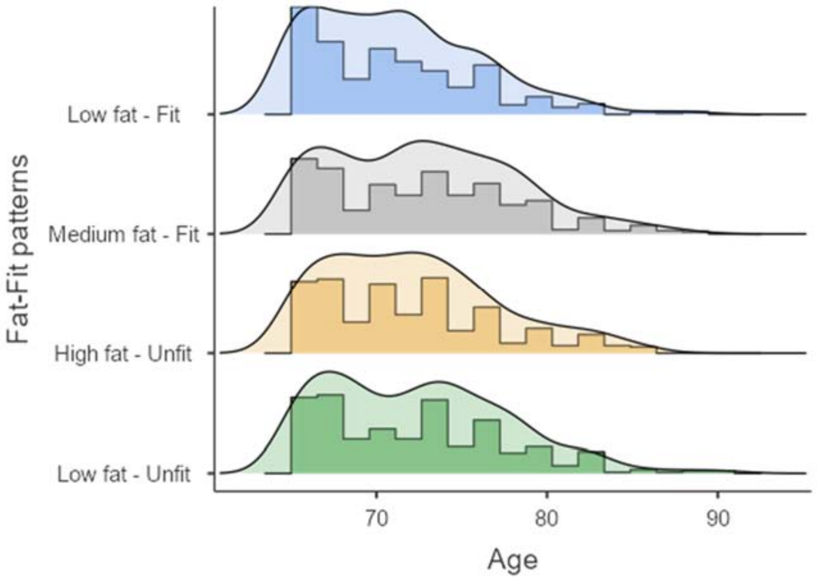

Figure S2. Distribution of sex in the four Fat-Fit patterns.

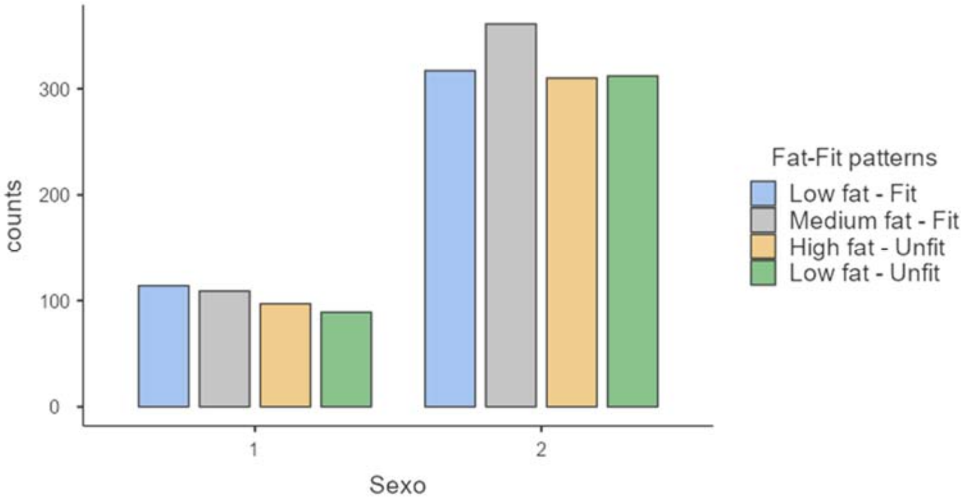

Figure S3. Distribution of fat percentage in the four Fat-Fit patterns.

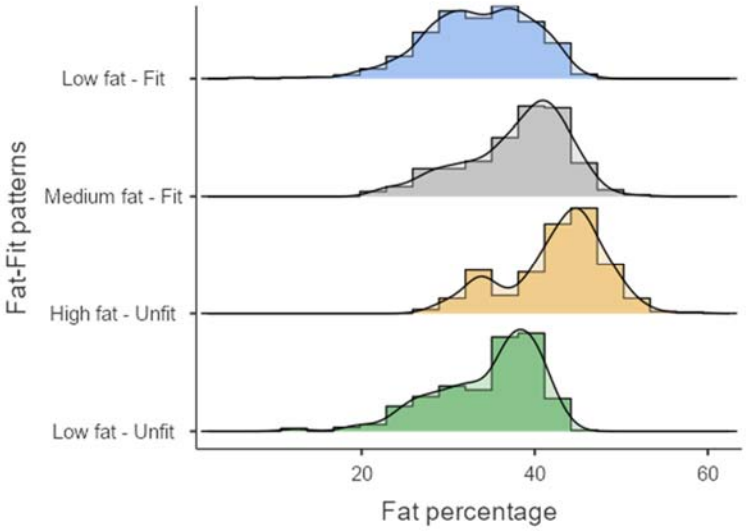

Figure S4. Distribution of BMI in the four Fat-Fit patterns.

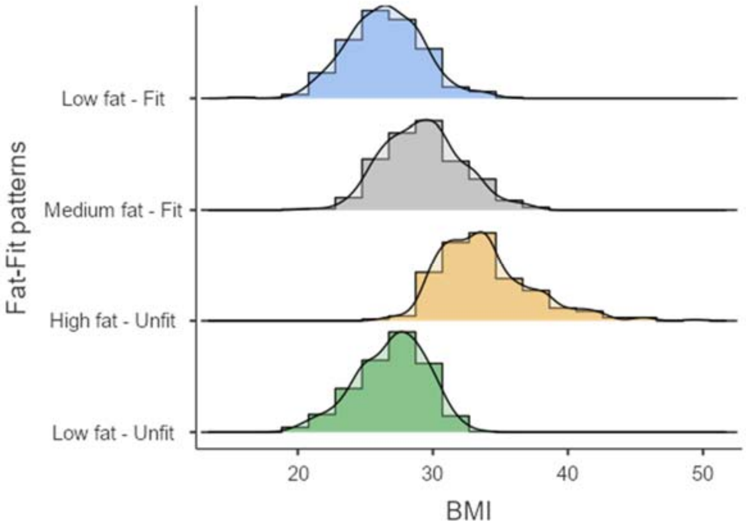

Supplement: Supplementary file 1 [file nutrients-13-02872-s001.zip › nutrients-1315023-supplementary.pdf]
